# Ion Release from Stainless Steel Brackets Subjected to Tooth Bleaching Treatment

**DOI:** 10.30476/dentjods.2022.95210.1854

**Published:** 2023-09

**Authors:** Ahmadreza Sardarian, Fatemeh Abbasi, Maryam Pakniyat Jahromi

**Affiliations:** 1 Orthodontics Research Center, Dept. of Orthodontics, School of Dentistry, Shiraz University of Medical Sciences, Shiraz, Iran; 2 Dept. of Oral and Maxillofacial Radiology, School of Dentistry, Shahid Sadoughi University of Medical Sciences, Tehran, Iran; 3 Student Research Committee, Postgraduate Student, Dept. of Pediatric Dentistry, School of Dentistry, Shiraz University of Medical Sciences, Shiraz, Iran

**Keywords:** Ion release, Orthodontic brackets, Home bleaching, Office bleaching

## Abstract

**Statement of the Problem:**

Tooth discoloration in the form of staining is a common finding during conventional orthodontic treatment. Due to elevated esthetic standards, clinicians in the field of orthodontics are faced with increasing demands by patients to perform bleaching treatments while the appliances are still in place. Though the success of such treatments has been reported in literature, the effect of whitening agents on orthodontic appliances has not been evaluated. Increased ion release following corrosion of orthodontic brackets is considered a health hazard.

**Purpose:**

In this study, we measured the amount of ion release from steel brackets under home and office bleaching treatment in order to evaluate the safety of such treatments during orthodontic therapy.

**Materials and Method:**

In this experimental study, a total of 120 brackets were randomly divided into 3 groups. The first group was subjected to an office bleaching regimen (hydrogen peroxide 40%). The second group was subjected to a home bleaching treatment (carbamide peroxide 20%). The third group did not receive any bleaching treatment. The specimens of all groups were immersed in artificial saliva and after 30 days, the amount of released chromium, copper, iron, magnesium, and nickel ions was measured and compared using one way ANOVA.

**Results:**

The results showed that ion release was significantly different between the three groups (*p*Value >0.05). For the chromium, iron, magnesium, and Nickel ion release the order was as follows: no bleaching < home bleaching < office bleaching. However, the copper ion release was greater in the group that received home bleaching.

**Conclusion:**

Ion release was enhanced when bleaching treatments were performed, with office bleaching having a more significant effect. Although the amounts of released ions were less than those permitted by WHO, we suggest that the clinicians recommend home bleaching for orthodontic patients that are seeking tooth whitening treatment.

## Introduction

Due to elevated life standards, there has been an increase in the demand for esthetic treatments. There are many esthetic treatments in dentistry including composite and ceramic veneers, full ceramic, porcelain fused to metal crowns, and tooth bleaching treatments. Bleaching discolored anterior teeth is one of the most popular esthetic dental treatments [ 1 ]. This treatment in combination with orthodontic and restorative treatments can help restore the shape, esthetics, and function of the tooth [ 2 ]. Tooth discoloration, is a common temporary side effect of orthodontic treatment and is due to the reduced capability of patients to keep their teeth clean during orthodontic treatments. There are increasing reports of patients seeking treatments to reduce discoloration during orthodontic therapy [ 3 ]. Various bleaching materials containing active hydrogen compounds with different concentrations are available for use in cosmetic dentistry. They are classified into two main categories of home (10-30% carbamide peroxide) and office (3-38% hydrogen peroxide) [ 3 ].

Orthodontic brackets are made of different materials. Despite the popularity of ceramic braces and clear aligners, metal brackets have kept their status as the gold standard in conventional orthodontic treatment [ 4 ]. Orthodontists can use braces and wires made from various alloys like stainless steel (iron, chromium, nickel), titanium, and cobalt-chromium. Due to its superior mechanical properties, stainless steel is the most commonly used alloy in orthodontic appliances [ 4 ].

Stainless steel brackets can release various ions into saliva among which nickel, chromium, iron, magnesium, and copper are the most important [ 5 ]. The adverse effects of nickel including carcinogenicity, irritability, and mutagenicity have been studied at the level of cell, tissue, organ, and organism [ 5 - 7 ]. Nickel ions are related to hypersensitivity, contact dermatitis, asthma, and cellular toxicity [ 8 ]. Chromium ion elevation and its accumulation in the body cause defects in bone formation, decreased body, and mandibular growth [ 9 ]. Excessive iron can lead to colorectal, renal, and hepatic malignancies. Furthermore, a study reported a higher rate of lung cancer incidence among people working in iron mines [ 10 ]. Excessive magnesium causes neural toxicity [ 11 ]. In addition, elevated level of copper increases the oxidative injury to lipids, proteins, and deoxynucleic acids and consequently lead to degenerative neurological diseases [ 12 ].

There are reports of the efficacy of bleaching treatment during orthodontic treatment [ 13 - 16 ], however, there are no studies investigating the effect of such treatments on ion- release from orthodontic appliances. In this study we aim to investigate the amount of ion release following treatment with two different regimens (home and office).

## Materials and Method

This experimental study (IR.SUMS.DENTAL.REC. 1398.082) was carried out in the Dental School of Shiraz University of Medical Sciences. Three groups of forty brackets (Mini-master Series, American Orthodontics, USA) were tested. The number of specimens in each group was chosen according to a study by Momeni *et al*. [ 13 ], which investigated ion release following immersion in conventional mouthwashes [ 13 ]. A 17*25 wire and a segment of ligature wire were added to each sample to simulate clinical conditions and take into account the alloy differences and the possibility of galvanic corrosion. The first and second group received in-office and home bleaching, respectively. The third group was considered the control group and was not in contact with any bleaching substances.

In-office bleaching was performed using 40% hydrogen peroxide (Ultradent Products, Inc. South Jordan, USA). According to the factory instructions, dentist should use this product for 3 sessions with at least a five-day interval and in each session the material can be used 3 times for a maximum 20 minutes. Therefore, the required time for this treatment was calculated to be 180 minutes (3 hours). The bleaching material was spread on a petri dish, and then the brackets, wire, and ligature wire were added. The brackets were placed face down so that their outer surface would be thoroughly in contact with the bleaching agent and the base was left untouched. This was done to better resemble the clinical conditions of the mouth. After three hours, the brackets and wires were rinsed to remove any remaining bleaching substance.

Home bleaching was performed using carbamide peroxide 20% (Ultradent Products, Inc. South Jordan, USA). According to the manufacturer’s instructions, this material was supposed to be used for 2-4 hours daily by the patient. According to a 2014 study by Lunardi *et al*. [ 14 ], this material was used 4 hours a day for 21 days. Therefore, the anticipated treatment time was 84 hours. The specimens were placed in a petri dish containing carbamide peroxide 20% just like the first group. After 84 hours, the specimens were rinsed.

In order to simulate the oral environment, all specimens were immersed in 20ml of artificial saliva (Table 1) and thermocycling was performed for 250 times. Subsequently, all brackets were placed in an incubator at 37˚C for 30 days. After this period, the specimens were removed from the saliva and the saliva was sent for measurement of the concentration of nickel, chromium, magnesium, copper, and iron ions. The concentrations were measured using inductively coupled plasma spectrometer (ICP-OES, Varian, Vista-Pro model, Mulgrave, Victoria, Australia; applied power: 1400W). The amount of ion released were compared between the three groups by using One way ANOVA test and Tukey post-hoc test.

**Table 1:** Ingredients of Bleaching gels and artificial saliva

Bleaching gel	Ingredients
Opalescence Boost 40%	Glycerin, water, Carbamide Peroxide, Xylitol, Carbomer, PEG-300, sodium hydroxide, EDTA, potassium nitrate, sodium fluoride
Opalescence PF20% regular	Glycerin, water, hydrogen Peroxide, Xylitol, Carbomer, PEG-300, sodium hydroxide, EDTA, potassium nitrate, sodium fluoride
Artificial saliva	Potassium chloride, sodium chloride, dipotassium hydrogen phosphate, potassium dihydrogen phosphate, sodium benzoate, sodium fluoride, phenyl mercuric nitrate, sodium carboxymethylcellulose, sorbitol solution, distilled water

## Results

The average, range and standard deviation of ion release from brackets of all three study groups are collected in the following Tables 1-6. According to the information resulting from data analysis using ANOVA and Tukey post-HOC tests, the amount of chromium, copper, iron, magnesium and nickel ions released was significantly different among the three groups (*p*< 0.001). The release of all ions from brackets and wires treated with in-office bleaching (hydrogen peroxide 40%) was more than the ones treated at home (carbamide peroxide 20%). Furthermore, the control specimens demonstrated the least amount of ion release except for copper ions. The copper ion release from the home bleaching group was lower than the control group.

**Table 2:** Concentration (μg/L) of chromium ion released in artificial saliva at 37˚C after 30 days: average concentration, standard deviation and range

Group	Number	Mean (μg/L)	Standard deviation	Confidence Interval 95%	
				Lower limit	Upper limit
Office bleaching	40	169.59 A	9.42	166.57	172.60
Home bleaching	40	18.94 B	6.49	16.86	21.01
Control	40	7.26 C	4.23	5.90	8.61

**Table 3:** concentration (μg/L) of copper ion released in artificial saliva at 37˚C after 30 days: average concentration, standard deviation and range

Group	Number	Mean (μg/L)	Standard deviation	Confidence Interval 95%	
				Lower limit	Upper limit
Office bleaching	40	108.97 A	13.22	104.74	113.21
Home bleaching	40	26.08 B	5.90	24.20	27.97
Control	40	50.58 C	7.23	48.27	52.90

**Table 4:** Concentration (μg/L) of Iron ion released in artificial saliva at 37˚C after 30 days: average concentration, standard deviation and range

Group	Number	Mean (μg/ L)	Standard deviation	Confidence Interval 95%	
				Lower limit	Upper limit
Office bleaching	40	1806.78A	256.24	1721.96	1891.61
Home bleaching	40	391.93B	90.15	363.09	420.76
Control	40	64.83 C	4.60	63.35	66.30

**Table 5:** Concentration (μg/L) of magnesium ion released in artificial saliva at 37˚C after 30 days: average concentration, standard deviation and range

Group	Number	Mean (μg/L)	Standard deviation	Confidence Interval 95%	
				Lower limit	Upper limit
Office bleaching	40	33.36 A	5.82	31.49	35.22
Home bleaching	40	25.64 B	12.21	21.73	29.54
Control	40	21.08 C	3.66	19.90	22.25

**Table 6:** Concentration (μg/L) of nickel ion released in artificial saliva at 37˚C after 40 days: average concentration, standard deviation and range

Group	Number	Mean (μg/ L)	Standard deviation	Confidence Interval 95%	
				Lower limit	Upper limit
In office bleaching	40	156.99 A	23.56	149.45	164.52
Home bleaching	40	109.97 B	29.54	100.52	119.41
Control	40	94.58 C	10.09	91.35	97.80

## Discussion

Increased demand for esthetics in the modern era has forced dentists to use different methods to improve the appearance of teeth. One of these procedures is bleaching, which is performed by using different materials and methods. Patients under orthodontic treatment have had complaints regarding tooth discoloration during the course of the treatment, and new research has proven whitening agents to be effective in these patients [ 15 ].

In this study, we evaluated the effect of two common home and office bleaching treatments considering the release of chromium, copper, iron, magnesium, and nickel ions from stainless steel brackets and wires used in orthodontic treatments. These bleaching treatments were performed using carbamide peroxide 20% and hydrogen peroxide 40%, respectively. In all three groups, maxillary premolar brackets were used to avoid the possible effect of size and shape differences on the amount of ion release. These two specific bleaching materials were chosen due to their commercial availability and their popularity among dental practitioners. Brackets were placed upside down so that only the outer surface of them would be in contact with the material. This way of interaction between bracket base and bleaching gel did not lead to excessive ion release and therefore, there was no bias in the results. After performing bleaching 

treatment according to manufacturer’s instructions, bleached brackets were immersed in artificial saliva before receiving thermocycling. Thermocycling simulates the oral situations with higher and lower temperatures.

The amount of ion release from specimens treated with in-office bleaching was significantly greater than the ion release from home bleaching and control specimens. This difference was due to presence of elevated amount of unstable free radicals like hydroxyl radicals, para hydroxyl radicals, para hydroxyl anions, and superoxide anions in hydrogen peroxide 40% compared to carbamide peroxide 20%. Higher levels of reactive oxygen species in the first group led to greater corrosion of the stainless-steel brackets and wires and therefore, higher amounts of ions were released from those brackets. Furthermore, there is an abundance of Oxygen and Nitrogen in carbamide peroxide, which acts as electron centers. These centers are present on the surface of the alloy and form a protective layer that hinders the surface corrosion [ 18 ]. Multiple studies have investigated the effect of bleaching materials and their concentrations on the corrosion of different dental alloys [ 18 - 23 ].

Comparing the control group with the home bleaching group, we realized that all of the measured ions (except for copper) were released in greater amounts from the second group. Interestingly copper ion measured in the control group was higher compared to the second group, which can be the result of the reaction between copper ions and carbamide peroxide. The urea present in carbamide peroxide has a protein structure, which can form a complex with copper. As a result, it is possible that these complexes had precipitated on the bracket surfaces and were taken out of the artificial saliva and therefore not measured. In agreement with this finding, a study conducted by Al-Salehi *et al*. [ 20 ] on the effect of carbamide peroxide concentration on dental amalgam alloy corrosion, the amount of copper ion release from the control group was higher than the amount of ion released from the carbamide peroxide 10% group [ 20 ].

We consume various types of drinks and foods every day through which different amounts of ions enter our bodies [ 24 ]. For example, the amounts of chromium and nickel ions entering the body are 5-100 and 300-500 mg, respectively. In addition, the concentration of nickel in drinking water is below 20μg/L while its chromium concentration is 0.43μg/L [ 17 ]. The maximum recommended daily amounts of edible nickel and chromium are 200-300 mg and 50-200 mg, respectively. The upper limit for oral copper intake by adults is 10 mg per day. This number for iron and magnesium intake is 20 and 5 mg per day [ 14 ]. Comparing the results of this study with the maximum amount recommended by WHO is difficult due to differences in applied units. However, the amount of ion release from all three groups was lower than their maximum permitted oral intake. The amounts of nickel and chromium ions released from the first and second group were closer to their maximum permitted intake compared to the other three ions [ 24 ].

From a general perspective, among all ions released from stainless steel, nickel and chromium ions are the main concern due to their high potential of allergic, toxic, and carcinogenic reactions [ 25 - 28 ]. Dental clinicians must be aware that metal ion release can cause local sensitivity reactions like redness with or without swelling in oral soft tissues. Furthermore, apart from poor oral hygiene, acute gingival inflammation can also be related to hypersensitivity reaction to nickel and chromium ions released from stainless steel alloy [ 29 ]. Therefore, it is very important that the practitioner evaluate patient’s history of hypersensitivity. Although the amount of ions released form orthodontic appliances is not significant, these appliances are present in the oral cavity for a long time throughout the treatment and can therefore cause various reactions.

Since there have not been any studies on this particular subject, comparing the results with previous studies is not possible. However, our method is very similar to a 2011 study conducted by Momeni *et al*. [ 17 ] on the ion release from stainless steel brackets as a result of using various mouthwashes [ 17 ]. In this study, ion release from stainless steel brackets was calculated in static conditions while in clinical conditions, greater amounts of ions may be released. This is because of the fact that consuming food and beverages (with lower pH) and dental plaque accumulation can increase metal corrosion. Furthermore, elimination of the protective oxidative layer by routine daily brushing can exacerbate the problem [ 24 ].

## Conclusion

According to this study, ion release under the effect of 40% hydrogen peroxide is higher than 20% carbamide peroxide. Although these amounts are lower than the maximum amounts recommended by WHO, it is better to recommend home bleaching methods for patients with fixed orthodontic appliances.

## Conflict of Interest

The authors declare that they have no conflict of interest.
